# Elucidating the molecular mechanisms underlying anti-inflammatory effects of *Morchella esculenta* in the arachidonic acid metabolic pathway by network pharmacology and molecular docking

**DOI:** 10.1038/s41598-023-42658-1

**Published:** 2023-09-23

**Authors:** Ma Xiaoying, Huo Zhiming, Yang Tao, Xiao Jun, Zhao Ying, Gong Na, Chen Xun, Liu Guoli, Wang Hong

**Affiliations:** 1 The Institute of Edible Fungi, Liaoning Academy of Agricultural Sciences, Shenyang, 110161 China; 2 Information Center, Guidaojiaotong Polytechnic Institute, Shenyang, 110161 China

**Keywords:** Computational biology and bioinformatics, Drug discovery, Immunology, Plant sciences, Biogeochemistry

## Abstract

*Morchella esculenta i*s an edible fungus with a uniquely delicious flavor and remarkable benefits for health. Herein, the molecular mechanism underlying the anti-inflammatory effects of *Morchella esculenta* was elucidated using molecular docking and network pharmacology. NPASS, Super-pred, SEA, Swiss Target Prediction, GeneCards, DisGeNET, Omim database, and STRING platform were used to select anti-inflammatory targets and construct target protein interaction networks using the active ingredients of *Morchella esculenta*. The OmicShare cloud platform was used to analyze GO functions and KEGG pathways related to the target, and the AutoDock Vina software was used to perform molecular docking and molecular dynamics (MD) simulation on the main target. Based on Cytoscape’s “Network Analysis”, the degree was used to identify potential key targets, and different inflammatory transcriptome data sets were used to evaluate core targets showing clinical significance. The active ingredient of *Morchella esculenta* identified from the NPASS database was EOYA, which had 43 anti-inflammatory targets, including NR1I2, PTGS1, PTGS2, CYP4F2, CYP3A4, TLR4, MAPK1, PLA2G4A, and PTPN11, and was mainly implicated in arachidonic acid metabolism, vascular endothelial growth factor signal pathway, and sphingomyelin signal transduction pathway, indicating that the anti-inflammatory effects of EOYA were mainly related to these biological processes. The degree was used to select 9 potential effective targets, namely NR1I2, PTGS1, PTGS2, CYP4F2, CYP3A4, TLR4, MAPK1, PLA2G4A, and PTPN11, among which NR1I2, PTGS1, PTGS2, PLA2G4A, MAPK1, CYP3A4, and TLR4 showed clinical significance. Molecular docking results showed that (E)-Octadec-11-En-9-Ynoic Acid (EOYA) could spontaneously bind to the 9 core targets, and the binding fractions of NR1I2, PTGS1, PTGS2, CYP4F2, and CYP3A4 were the highest. The MD simulation results showed that EYOA did indeed bind well NR1I2 to PTGS2, and the complex has high stability. *Morchella esculenta* can regulate the activity of prostaglandin endoperoxide synthetase, and affect the biosynthesis of prostaglandins, thereby impacting the metabolic pathway of arachidonic acid.

## Introduction

Inflammation, mediated by inflammatory cytokines and immune cells, is a defense response to injury, infection, allergy, and necrosis^1^. It is characterized by fever, redness, pain, and swelling^2^. Inflammatory responses are essential for defense against external stimuli and critical to the healing process^3^. However, chronic inflammation is associated with several diseases, including cancer, arthritis, cardiovascular disease, atherosclerosis, nervous system disorders, and autoimmune diseases^4^. Identifying promising non-toxic anti-inflammatory drugs is a target area of biomedical research. Aspirin, diclofenac, and other commonly used drugs for treating inflammation have serious side effects. Thus, anti-inflammatory agents based on natural products have become popular in recent years^5^.

*Morchella esculenta* is a delicious edible fungus with a unique flavor and remarkable health benefits^6^, and it contains various nutrients and exerts anti-oxidation, anti-inflammatory, and anti-tumor pharmacological activities^7^. *Morchella esculenta* produces several primary and secondary metabolites, including proteins, polysaccharides, sterols, polyphenols, alkaloids, fatty acids, flavonoids, and other small molecules^6^. A heteropolysaccharide isolated from the fruiting body of *Morchella esculenta* has an anti-inflammatory role as it blocks the activation of the NF-κB signaling pathway^8^. Simultaneously, total flavonoids of Morchella-fermented liquid show anti-inflammatory effects in RAW264.7 macrophages stimulated by lipopolysaccharide^9^. The hot water–ethanol extract of Morchella mycelia has an obvious inhibitory effect on acute and chronic inflammation^10^. EOYA is a natural octadecyl-conjugated enyne acid, wherein the special enyne-conjugated structure endows it with many biological activities, such as antibacterial^11^, antifungal^12^, larvicidal^13^ and anti-inflammatory^14,15^ properties.

In recent years, basic research on the active substances of traditional Chinese medicines has gained traction. To better clarify the pharmacological mechanisms underlying traditional Chinese medicine in the treatment of diseases, computational methods like molecular docking, network pharmacology, and dynamic simulation have emerged as effective tools^16,17^. At present, most of the researches on network pharmacology are Chinese herbal medicines and prescriptions^18–20^, but there are few studies on edible and medicinal fungi.The pharmacological activity of cordycepin, the active component of *Cordyceps militaris*, was studied by means of network pharmacology and molecular docking, and the core targets for improving Alzheimer's disease were screened^21^. However, there is no related research on *Morchella esculenta*.Herein, we employed network pharmacology to assess the anti-inflammatory targets of the active ingredient of *Morchella esculenta*, EOYA, and conducted protein interaction analysis, GO function analysis, and KEGG pathway analysis of these targets. We also used the GEO database to evaluate the clinical significance of the core targets and performed molecular docking and molecular dynamics (MD) simulation verification for the core targets to comprehensively elucidate the molecular mechanism underlying the anti-inflammatory effects of the active ingredient of *Morchella esculenta*.

### Target selection of active components from *Morchella esculenta*

In the NPASS database (http://bidd.group/NPASS/), “*Morchella esculenta*” was entered as the organism to search for the active ingredients of morel. In the Superpred (http://prediction.charite.de/), Swiss Target Prediction (http://swisstargetprediction.ch/), and SEA (http://sea.bkslab.org/) databases, the “SMILES” value of the active ingredient of *Morchella esculenta* was entered; the structure diagram of the substance was drawn, and the target of the active ingredient was assessed. The target of the active ingredient of *Morchella esculenta* was obtained by combining and removing the targets obtained from the above three databases.

### Selection of anti-inflammatory targets and construction of target network using the active components of *Morchella esculenta*

The keyword “anti-inflammatory” was entered in DisGeNET (www.disgenet.org/), GeneCards (www.genecards.org/), and Omim databases (https://www.omim.org/) to search for related targets, which were scored according to the photographic significance. Subsequently, the anti-inflammation targets of the three databases were combined to obtain significant anti-inflammation targets. The disease targets and the active ingredient targets of *Morchella esculenta* were input into Venny2.1.0 (https://bioinfogp.cnb.csic.es/tools/venny/), and the intersection of the two yielded the anti-inflammation targets of the active ingredient of *Morchella esculenta,* which were together imported into the Cytoscape 3.9.1 software to build the active ingredient_anti-inflammation target network.

### Construction of an anti-inflammatory target interaction network (PPI) using active components of *Morchella esculenta*

The anti-inflammatory targets of the active ingredient of *Morchella esculenta* obtained as described above were imported into STRING (www.string-db.org/) to construct the protein–protein interaction networks (PPI). The organism was set as “*Homo Sapiens*”; the minimum interaction threshold value was set at “medium confidence = 0.4”, and default values were retained for other parameters. The PPI network was imported into the Cytoscape 3.9.1 software, and the “cytoHubba” function was used to calculate the degree, betweenness, closeness, and other topological parameters for each network node. The greater the betweenness, closeness, and degree of the node, the more important the position of the node in the network. The targets with a degree, betweenness, and closeness above the corresponding average values were selected as the main anti-inflammation target of the active ingredient of *Morchella esculenta*.

### GO function and KEGG pathway enrichment analysis for anti-inflammatory targets of *Morchella esculenta*

The anti-inflammatory targets of the active ingredient of *Morchella esculenta* were analyzed by GO and KEEG analyses using the DAVID web tool (https://david.ncifcrf.gov/), and the obtained data were further analyzed by OmicShare (http://omicshare.com/) cloud platform for assessing dynamic GO enrichment. The UNIPROT website (www.uniprot.org/) was used to convert the anti-inflammatory targets of the active ingredient of *Morchella esculenta* into corresponding Gene Symbols, and the OmicShare cloud platform was used to conduct KEGG pathway (www.kegg.jp/kegg/kegg1.html) enrichment analysis for the anti-inflammatory targets of the active ingredient of *Morchella esculenta*.

### Clinical characteristics and tissue enrichment of key targets

The transcriptome data for inflammation were retrieved through the GEO database (https://www.ncbi.nlm.nih.gov/genome) to obtain the clinical significance of core targets. The differences in core target gene expression before and after inflammation were analyzed. Graphpad prism8 was used to draw a box chart. The Human eFP Browser (http://bar.utoronto.ca/efp_human/cgi-bin/efpWeb.cgi)^22^ was used to study the distribution of expression of the key targets, and the whole-body expression distribution map was obtained.

### Molecular docking verification of active components in *Morchella esculenta* and the major anti-inflammatory targets

The 2D structure of the active ingredient of *Morchella esculenta* was obtained from the PubChem database (https://pubchem.ncbi.nlm.nih.gov/), and the structure of ligand was sketched, hydrogens added, energy minimized and saved as mol2 files using the Chem3D software for storage. The target protein complex was searched in the PDB database (http://www.rcsb.org/), We manually curated the PDB to choose structures that described continuous, well resolved (resolution less than 2.5 Å), single-chain proteins and ligand binding. and the Discovery studio software was used to remove phosphate radicals, water molecules, and inactive ligands from the target protein. The target protein was imported into AutoDock^23^ Tools software for hydrogenation and charging. Subsequently, the target protein and active components of *Morchella esculenta* were introduced into the molecular docking website, CB-DOCK2 (CB-Dock2: An accurate protein–ligand blind docking tool (labshare.cn)) for molecular docking, and the docking combination with the lowest binding free energy was selected. The receptor-ligand binding image was visualized using the Pymol (2.5.0) software (www.pymol.org/)^24^.

### Molecular dynamics (MD) simulation

Molecular dynamics (MD) simulation of ligand-receptor docked complex EYOA-NR1I2 and EYOA-PTGS2 with the lowest Binding Free Energy were carried out using GROMACS^25^ (version 2021.2). Protein topology file was generated using the AMBER99SB-ILDN force field, whereas ligand topology file was generated by ACPYPE script using the AMBER fore field. MD simulation carried out in a triclinic box filled with TIP3 water molecules and periodic bounding conditions were applied. System was neutralized with NaCl counter ions. Before MD simulation, the complex was minimized for 1000 steps and equilibrated by running NVT and NPT for 100 ps. Then MD simulation was performed for 100 ns for each system under periodic boundary conditions at 310 K temperature and 1.0 bar pressure.

## Results

### Identification of the anti-inflammatory target of active ingredients in *Morchella esculenta*

**Figure 1:**
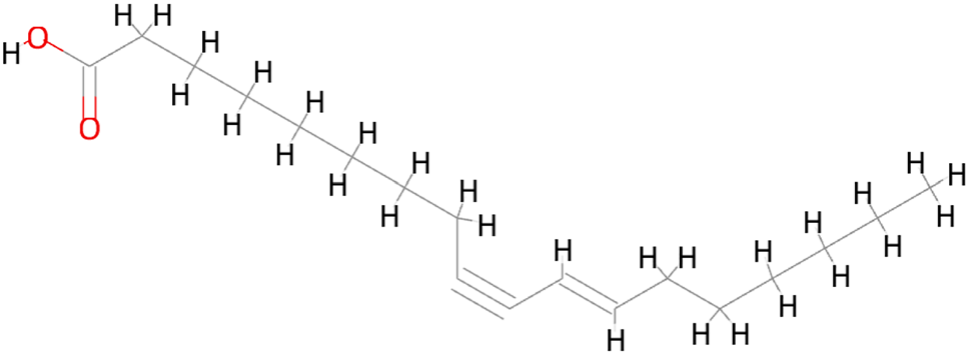
Chemical structure of the active ingredient of *Morchella esculenta*, EOYA.

**Figure 2:**
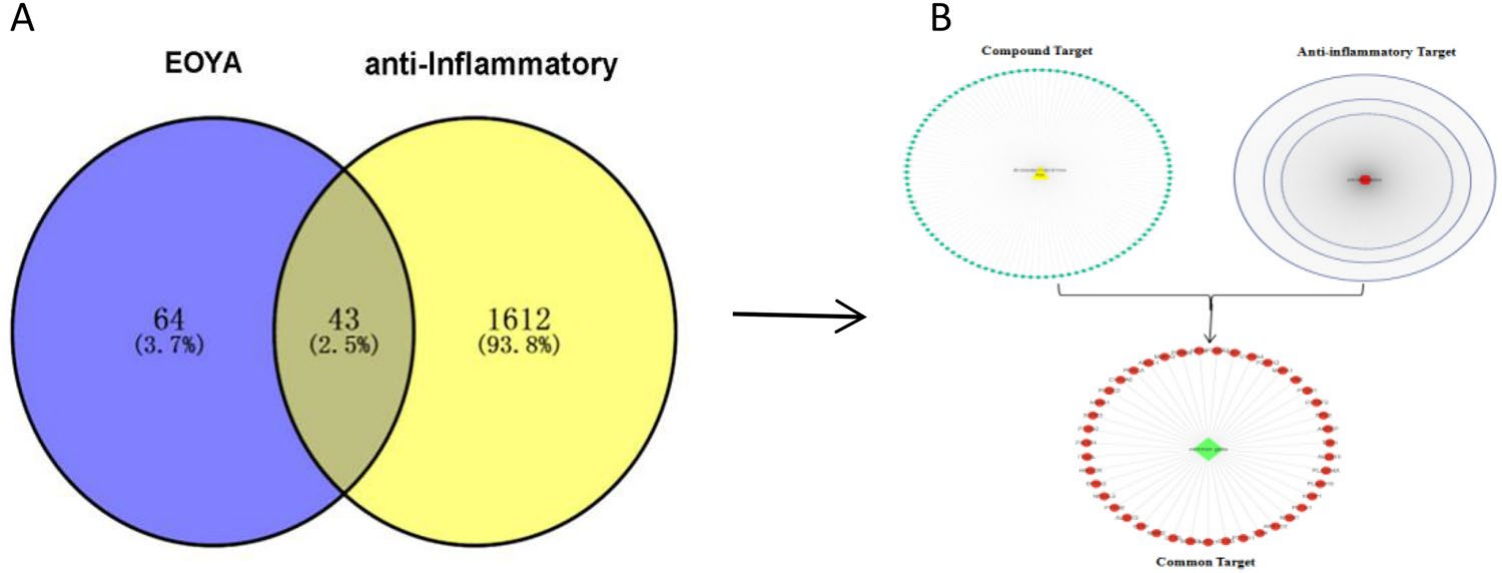
EOYA-anti-inflammatory Venn plot (**A**); EOYA-anti-inflammatory target network (**B**).

The active ingredient of “*Morchella esculenta*” according to the NPASS database was (E)-Octadec-11-En-9-Yinoic Acid (EOYA) (Fig. 1), numbered NPC179764, with a chemical formula of C_18_H_30_O_2_, and ‘SMILE’ value, CCCCCC/C=C/C#CCCCCCCCC(=O)O. The targets obtained by searching the “EOYA Smile Value” in the Super-pred, SEA, Swiss Target Prediction databases were merged and de-weighed, and 107 targets of EOYA were obtained. A total of 1654 anti-inflammation-related targets were obtained from GeneCards, DisGeNET, and Omim databases. The EOYA and anti-inflammatory targets were imported into the Venny 2.1.0, and 43 EOYA–anti-inflammatory targets were obtained after de-duplication (Fig. 2).

### Establishing the anti-inflammatory-target PPI Network using active components in *Morchella esculenta*

**Figure 3:**
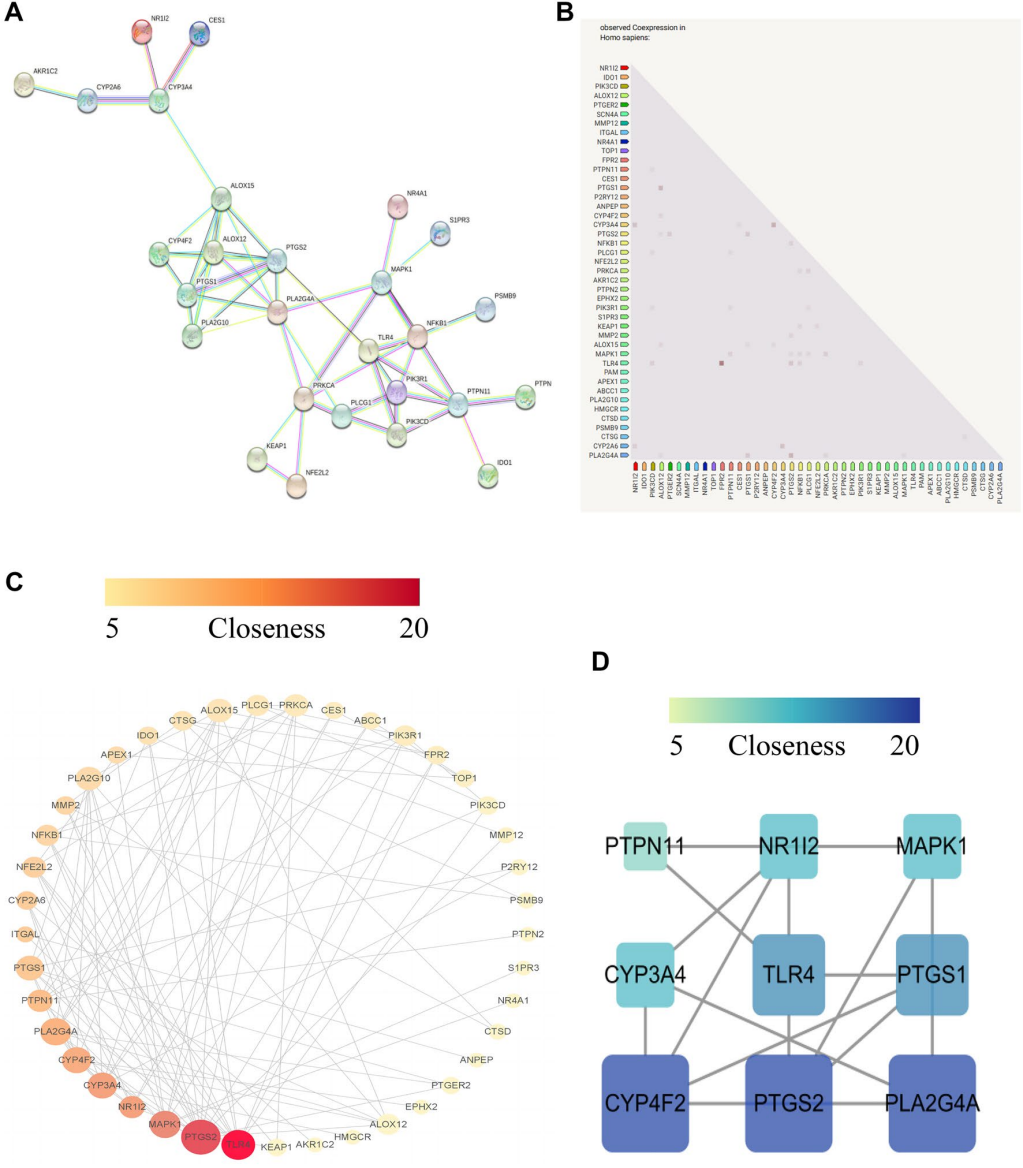
EOYA-anti-inflammatory target PPI network diagram (**A**); Co-expression heatmap of EOYA-anti-inflammatory targets (**B**); 43 EOYA-anti-inflammatory targets network (**C**); 9 key targets of EOYA-anti-inflammatory PPI network (**D**).

EOYA and its 43 anti-inflammation targets were imported into the Cytoscape 3.9.1 software to obtain the EOYA anti-inflammation target network (Fig. 3). The PPI network of anti-inflammatory targets is shown in Fig. 3. Each target participating in the interaction is represented by a circle. The larger the circle area, the greater its degree. The thicker the circle’s border, the greater betweenness. The darker the circle, the greater its closeness. There witsere 43 nodes and 104 interactions in the PPI network, with an average degree, betweenness, and closeness of 4.84, 59.63, and 17.70, respectively. The simultaneous measures of degree, betweenness, and closeness above the average yielded 9 targets (Table 1), indicating their key position in the PPI network and role as the main targets of EOYA responsible for its anti-inflammatory effect. Nine main targets, PTGS2, TLR4, MAPK1, PLA2G4A, CYP3A4, CYP4F2, PTGS1, NR1I2, and PTPN11 were analyzed. MAPK1, PTPN11, and TLR4 were involved in inflammation; PTGS1, PLA2G4A, CYP3A4, CYP4F2, PTGS1, and NR1I2 were involved in arachidonic acid-related pathways, and CYP3A4, PTGS2, NR1I2, and PTGS1 were involved in the synthesis of prostaglandins.

**Table 1:** Topological isomerization parameters of key anti-inflammatory targets of the active component of *Morchella esculenta.*

Target	Degree	Betweenness	Closeness
PTGS2	17	374.16	27.33
TLR4	13	430.65	25.50
MAPK1	11	254.53	23.33
PLA2G4A	11	129.10	24.08
CYP3A4	10	184.90	21.83
CYP4F2	10	151.02	22.67
PTGS1	9	82.92	22.42
NR1I2	7	185.80	21.67
PTPN11	7	99.92	20.42

### GO function and enrichment analysis for the anti-inflammatory target of *Morchella esculenta*

**Figure 4:**
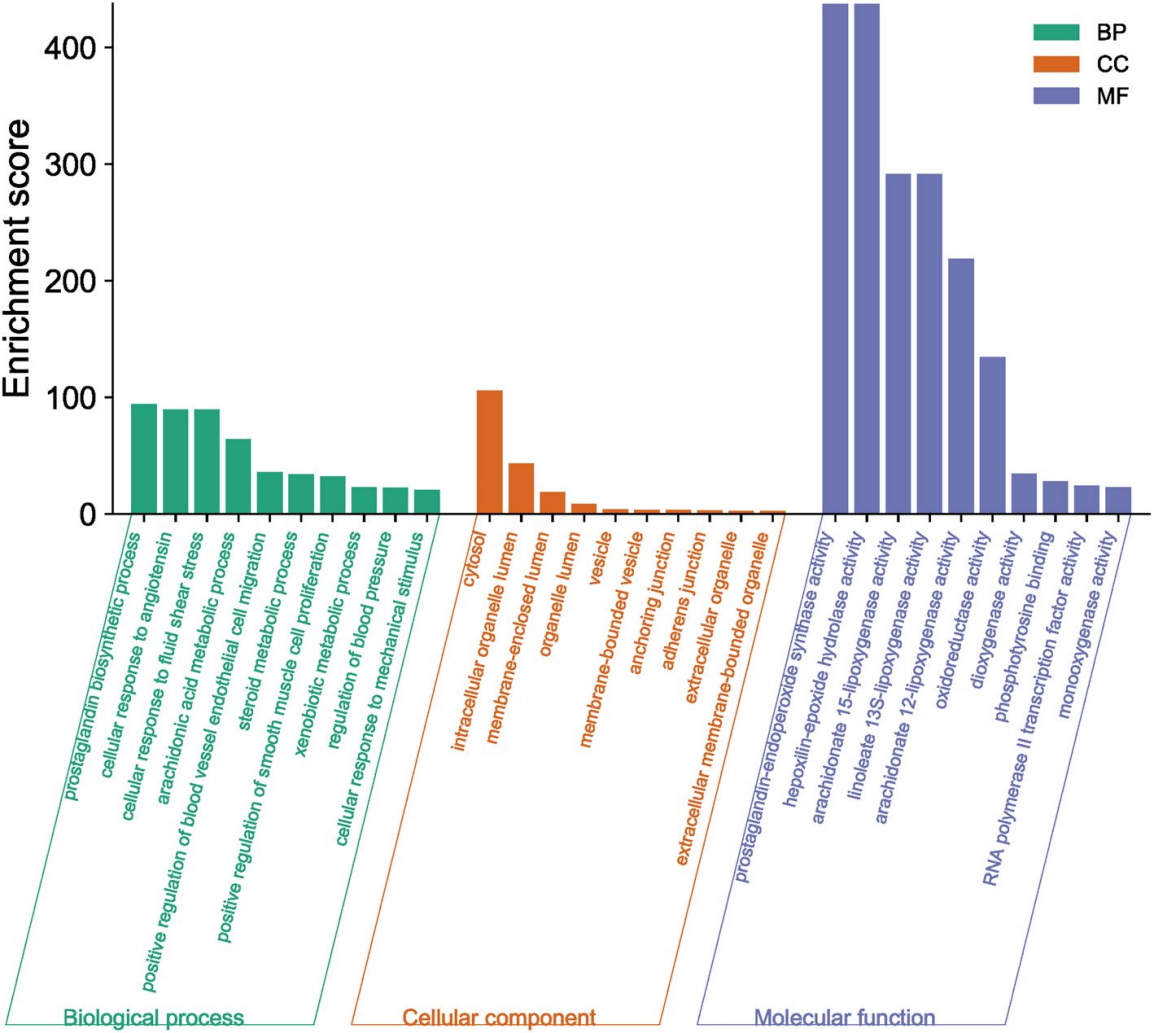
Enrichment analysis for the 43 anti-inflammatory targets of EOYA.

The DAVID database was used to obtain enrichment data for 43 targets, and the dynamic GO enrichment analysis based on the OmicShare cloud platform was used to select 322 GO entries with *P* < 0.05, including 22 molecular functions, 24 cellular components, and 276 biological processes. As shown in Fig. 4, biological processes mainly included biosynthesis, cell reaction, cell metabolism, and signal transduction; molecular functions included binding, enzyme activity, and regulation, and the cellular components included organelles, vesicles, and extracellular matrix. The target functions were enriched in prostaglandin biosynthesis, prostaglandin endoperoxide synthetase activity, and cell response to angioprogressive hormone, indicating that the anti-inflammatory effects of EOYA were mainly related to these biological processes.

### KEGG pathway enrichment analysis for the anti-inflammatory targets of the active component in *Morchella esculenta*

**Figure 5:**
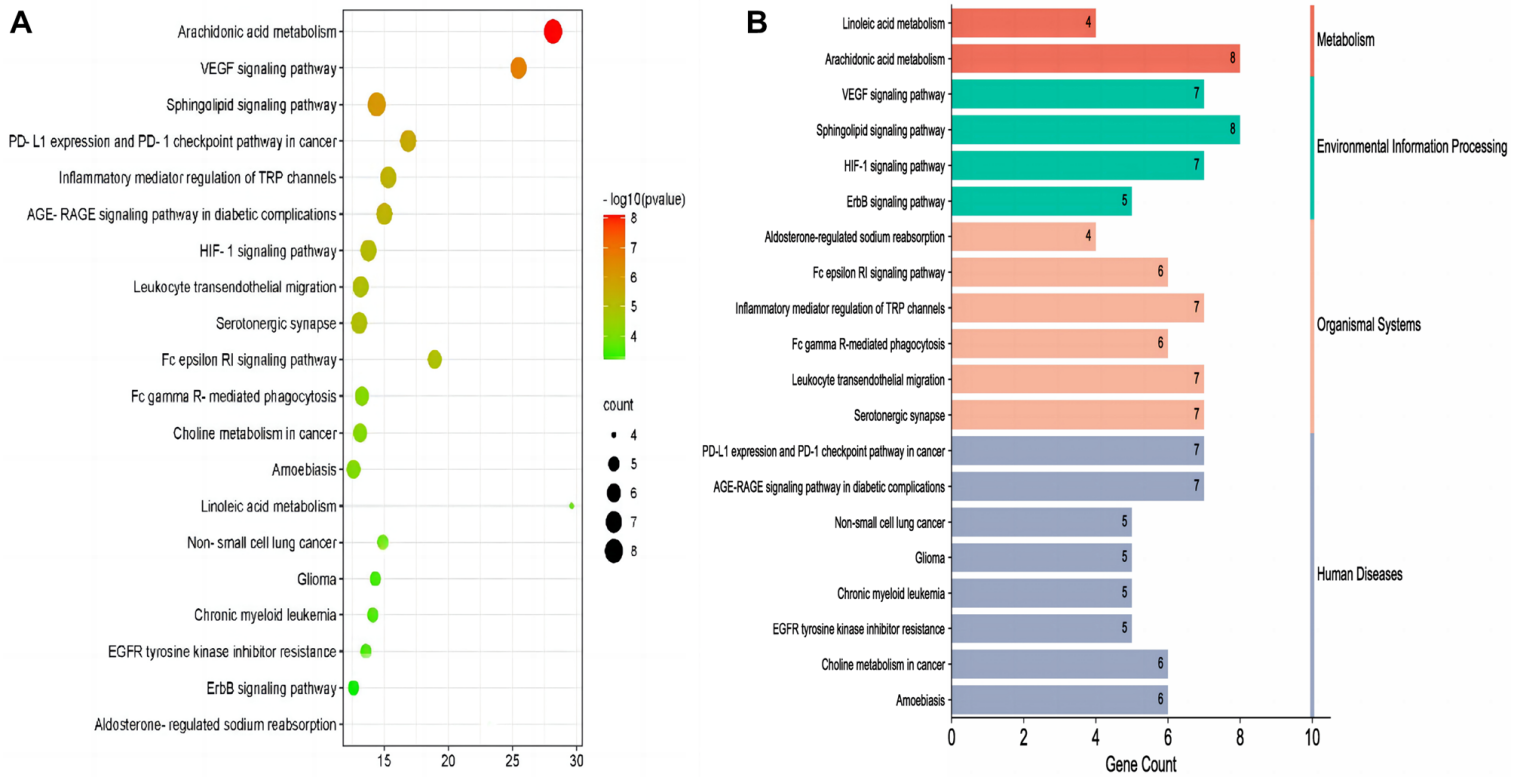
The Bubble plot of *KEGG* pathway analysis for EYOA-anti-inflammatory targets (**A**); The Histogram of *KEGG* pathway analysis for EYOA-anti-inflammatory targets (**B**).

**Figure 6:**
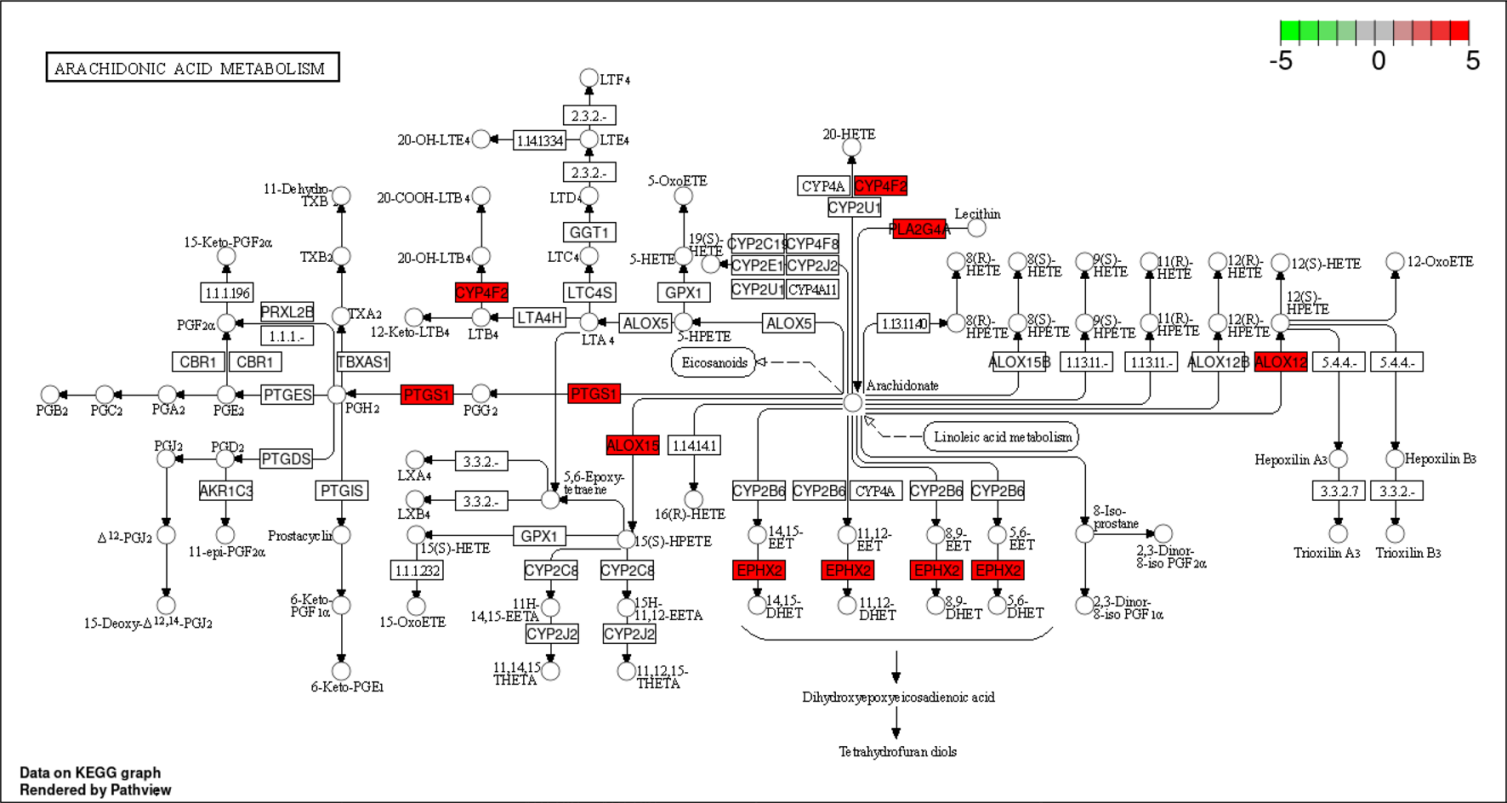
Related targets of the active ingredient of *Morchella esculenta* in the arachidonic acid metabolism (map00590) pathway.

Using *P* < 0.001, 20 KEGG pathways related to the anti-inflammatory targets of EOYA, were selected. Figure 5 shows the enrichment bubble diagram for each pathway. The enrichment fraction of the target was the degree of EOYA enrichment for the target in a pathway and all the involved proteins. The greater the value, the higher the enrichment degree. The color represents the *P*-value. The redder the color, the smaller the *P*-value, and the more significant the enrichment. The size of the point indicates the number of enrichment targets in the pathway. The larger the point, the more the number of enrichment targets. The pathways with large target values mainly included arachidonic acid metabolism, vascular endothelial growth factor signal pathway, and sphingomyelin signal transduction pathway. Among them, the arachidonic acid metabolism^26^ pathway (Fig. 6) showed the greatest number of enriched targets, the highest target value, and the lowest *P*-value.

### Expression of anti-inflammatory core genes targeted by the active components of *Morchella esculenta*

**Figure 7:**
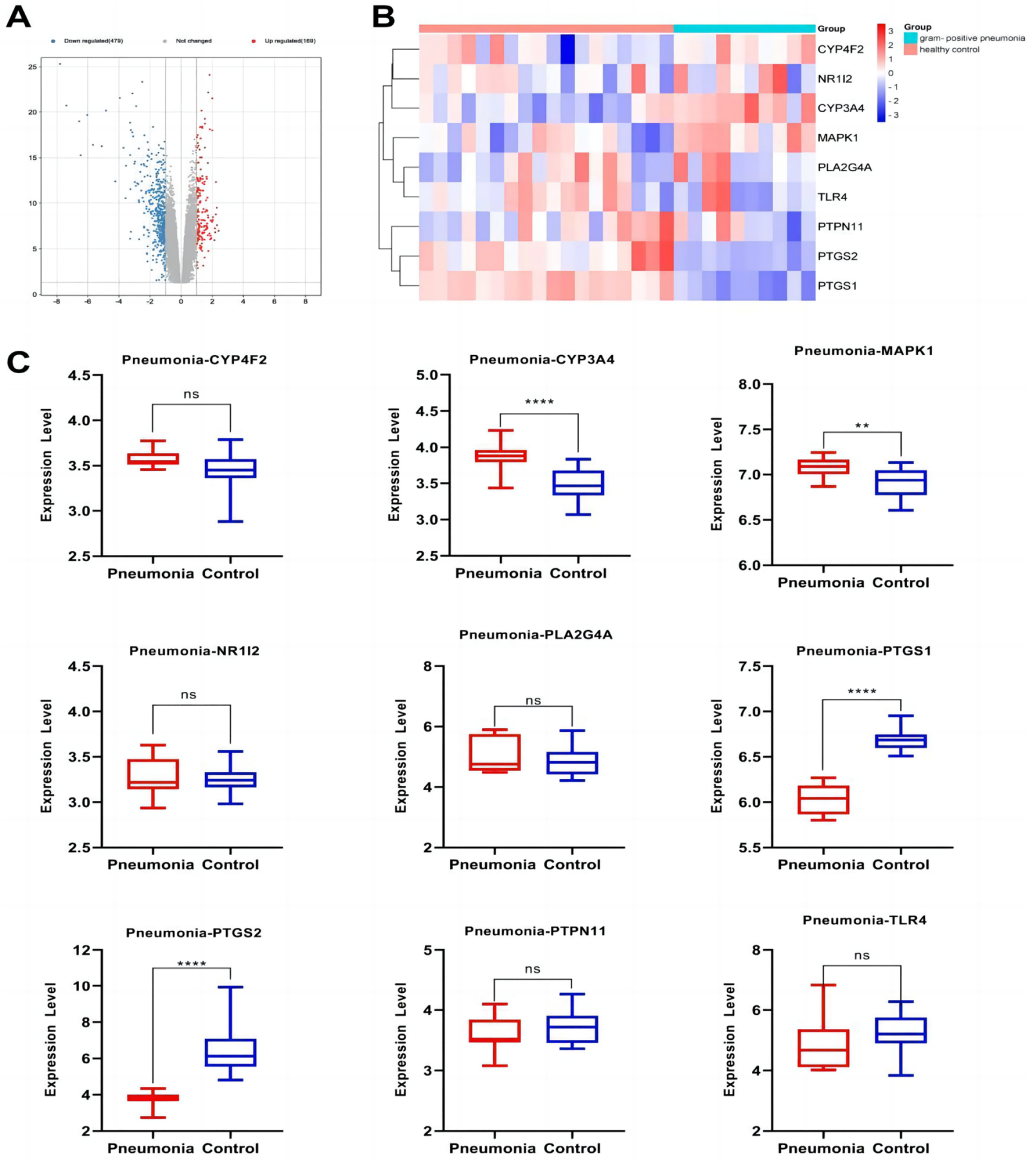
(**A**) mRNA expression analysis using the GEO dataset for pneumonia; (**B**) Composition-Target binding energy heat map; (**C**) mRNA expression analysis for the key targets of EOYA using the GEO database; *p < 0.05, **p < 0.01.

**Figure 8:**
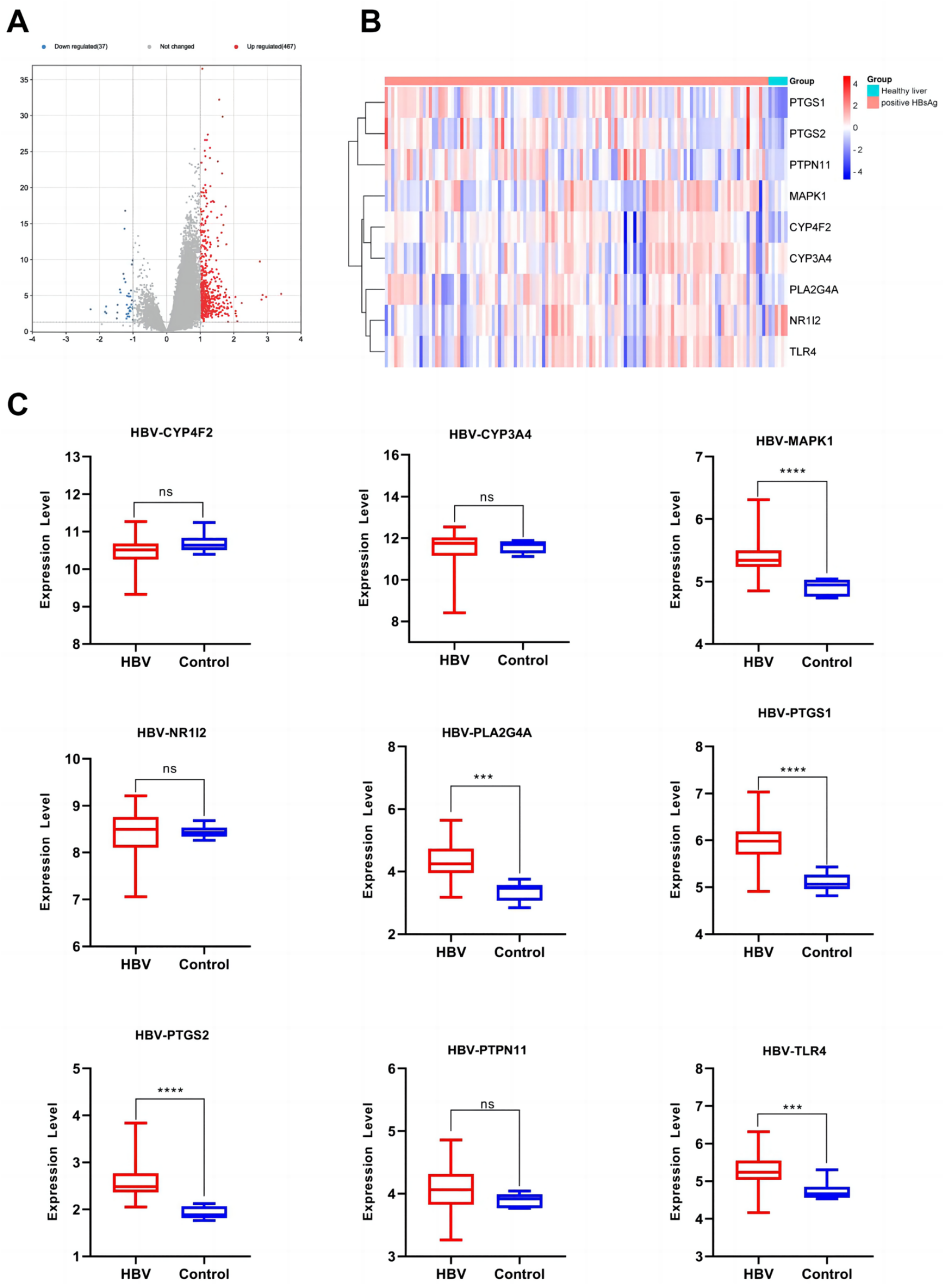
(**A**): mRNA expression analysis using the GEO dataset for hepatitis; (**B**): Composition-Target binding energy heat map. (**C**): mRNA expression analysis for the key targets of EOYA using the GEO database; *p < 0.05, **p < 0.01.

**Figure 9:**
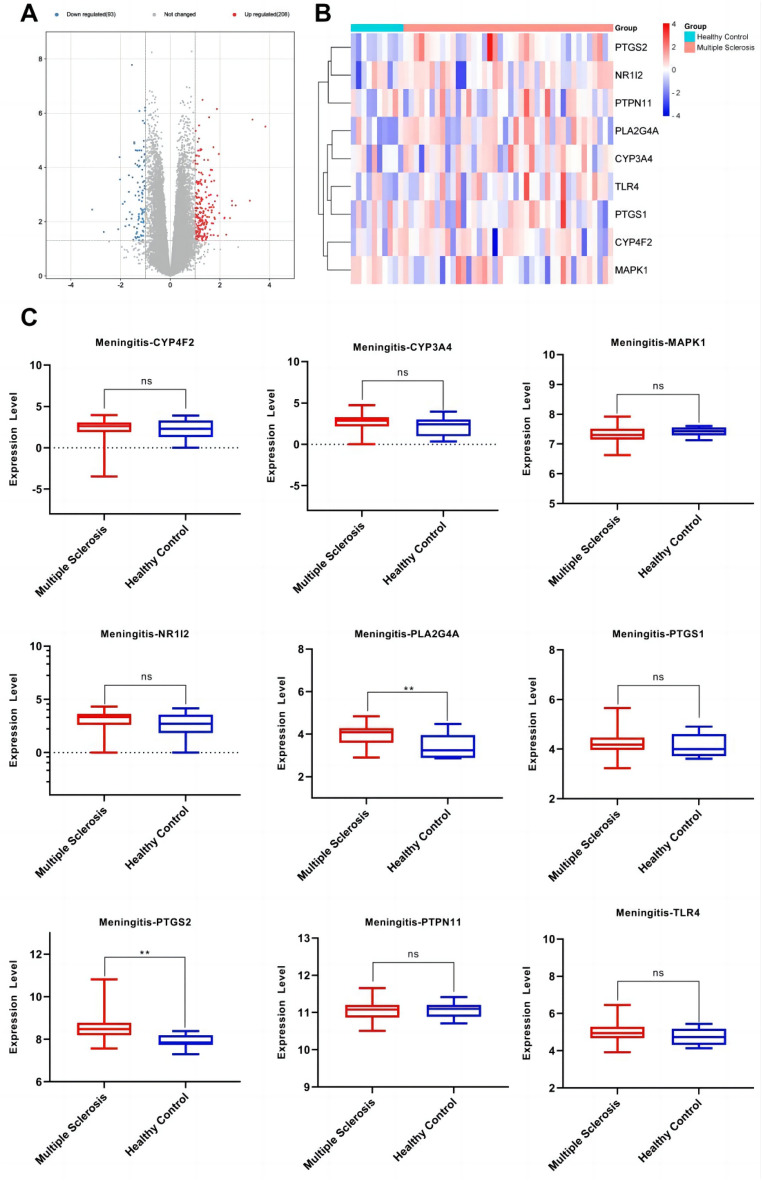
(**A**): mRNA expression analysis using the GEO dataset for meningitis. (**B**): Composition-Target binding energy heat map. (**C**): mRNA expression analysis for the key targets of EOYA using the GEO database; *p < 0.05, **p < 0.01.

**Figure 10:**
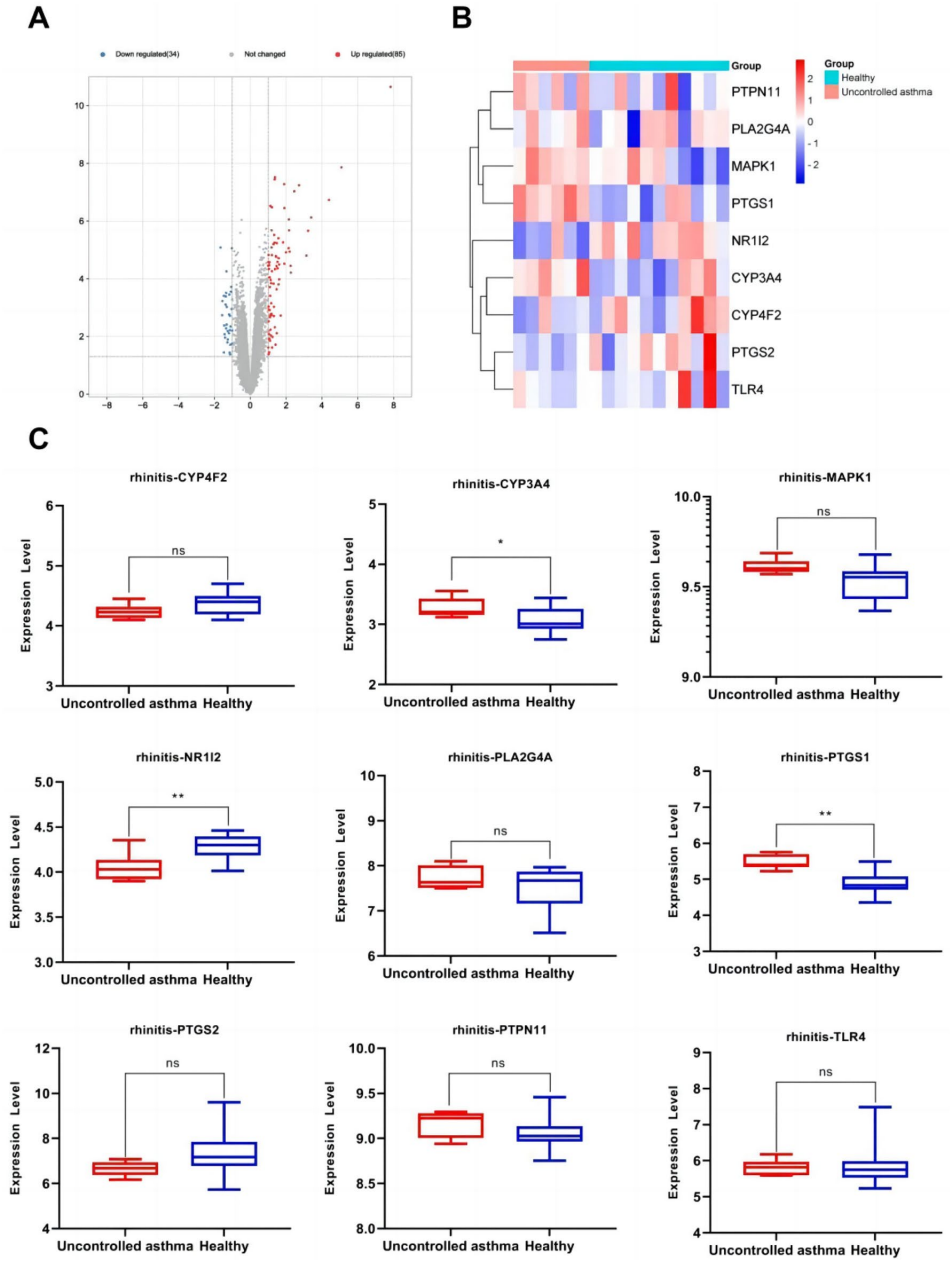
(**A**): mRNA expression analysis using the GEO dataset for rhinitis. (**B**): Composition-Target binding energy heat map. (**C**): mRNA expression analysis for the key targets of EOYA using the GEO database; *p < 0.05, **p < 0.01.

**Figure 11:**
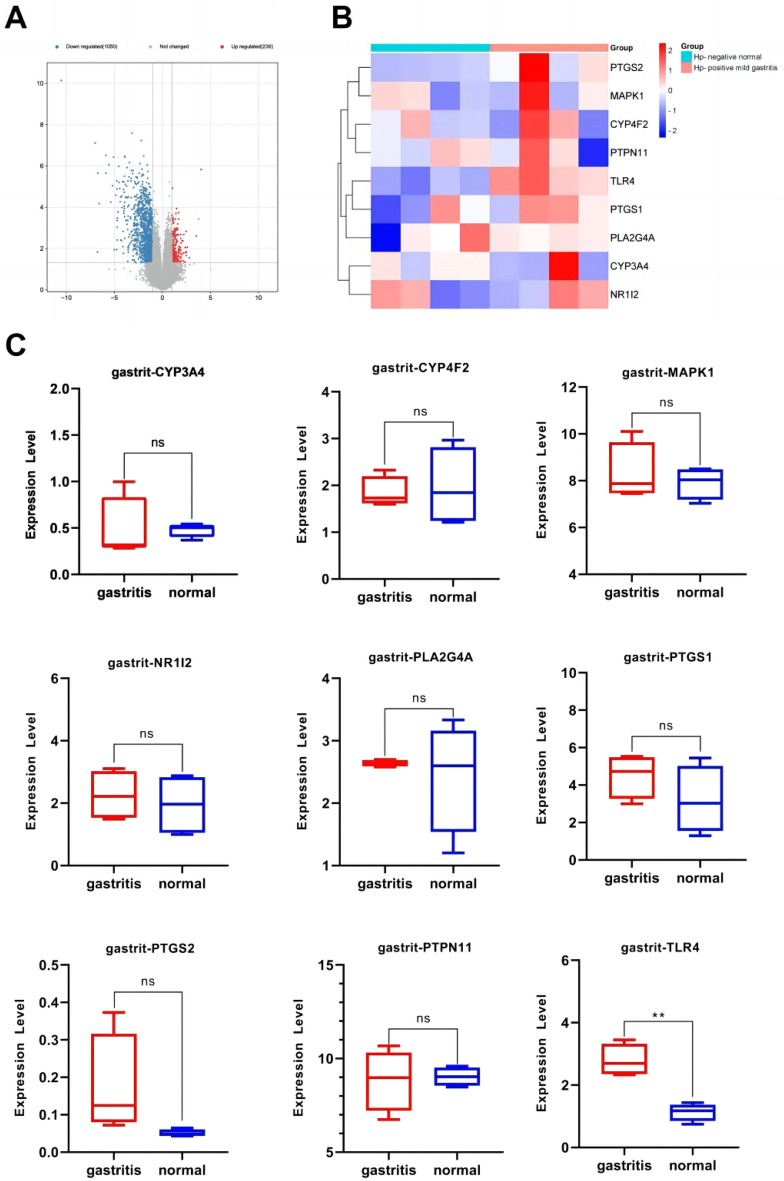
(**A**): mRNA expression analysis using the GEO dataset for gastritis. (**B**): Composition-target binding energy heat map. (**C**): mRNA expression analysis for the key targets of EOYA using the GEO database; *p < 0.05, **p < 0.01.

**Figure 12:**
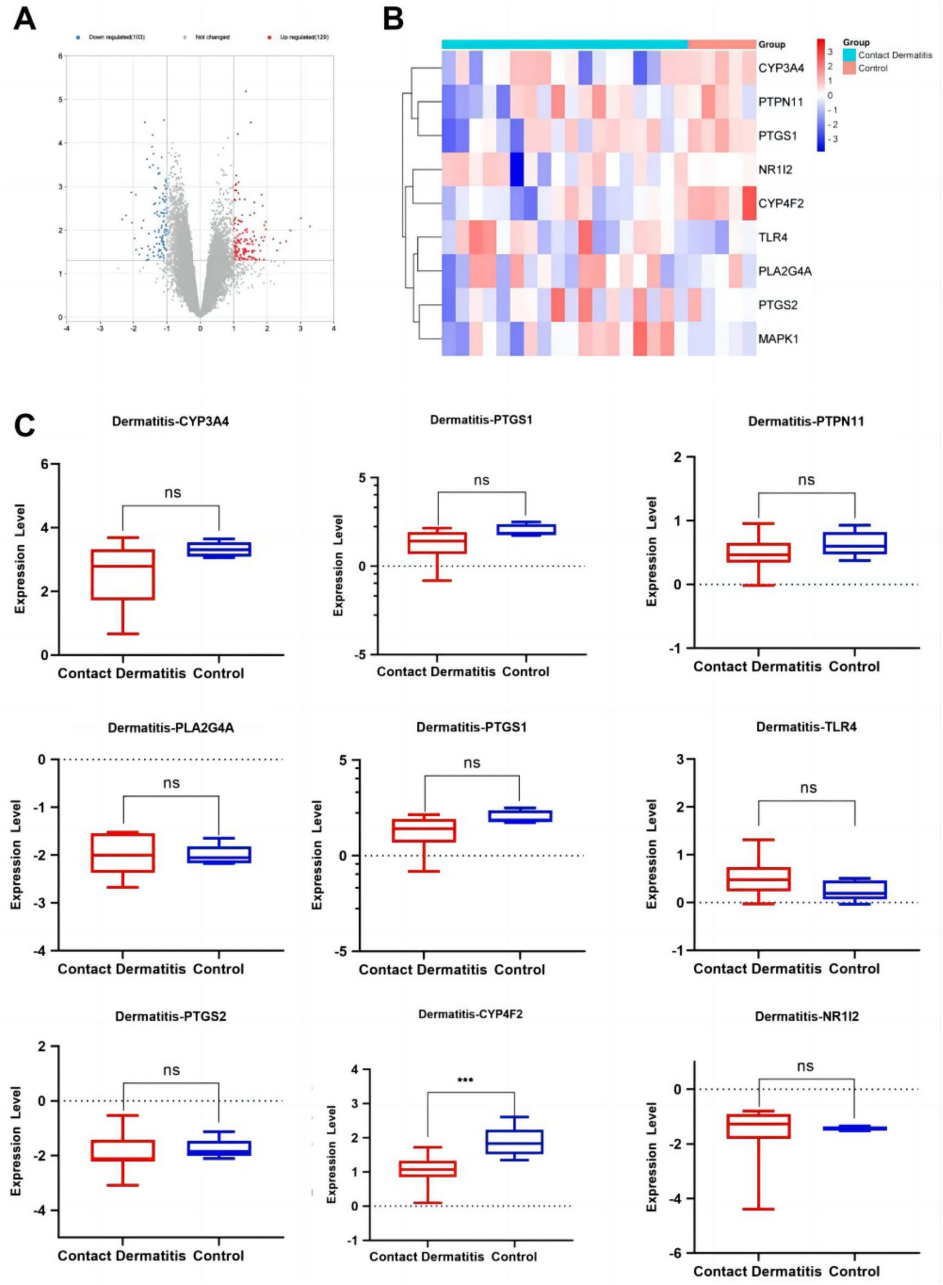
(**A**): mRNA expression analysis using the GEO dataset for dermatitis. (**B**): Composition-target binding energy heat map. (**C**): mRNA expression analysis for the key targets of EOYA using the GEO database; *p < 0.05, **p < 0.01.

The data set of inflammation was screened according to the significant difference in the expression of core targets. Using the transcription data of the dermatitis GSE153007 dataset, neuroinflammation GSE135511 dataset, rhinitis GSE19187 dataset, pneumonia GSE35716 dataset, hepatitis GSE83148 dataset, and gastritis GSE60427 dataset, the corresponding expressions of the nine core genes were analyzed. As shown in Figs. 7, 8, 9, 10, 11, 12, in the transcriptome data of contact dermatitis, the expression of CYP4F2 reduced significantly. The expressions of PTGS1 and CYP3A4 were markedly increased in patients with rhinitis accompanied by asthma, while the expression of NR1I2 decreased synonym. The mRNA expressions of PLA2G4A and PTGS2 in neuritis causing multiple cerebral infarctions increased significantly as compared to those in normal subjects. In cases of bacterial pneumonia, the mRNA expression of MAPK1 and CYP3A4 increased significantly, while those of PTGS1 and PTGS2 decreased synonym. In HBV-induced hepatitis, the expressions of MAPK1, PTGS1, PTGS2, PLA2G4A, and TLR4 increased significantly. On comparing the transcriptome of patients with gastric inflammation and normal individuals, mRNA expression of TLR4 was found to be synonym increased in the former group.

**Figure 13:**
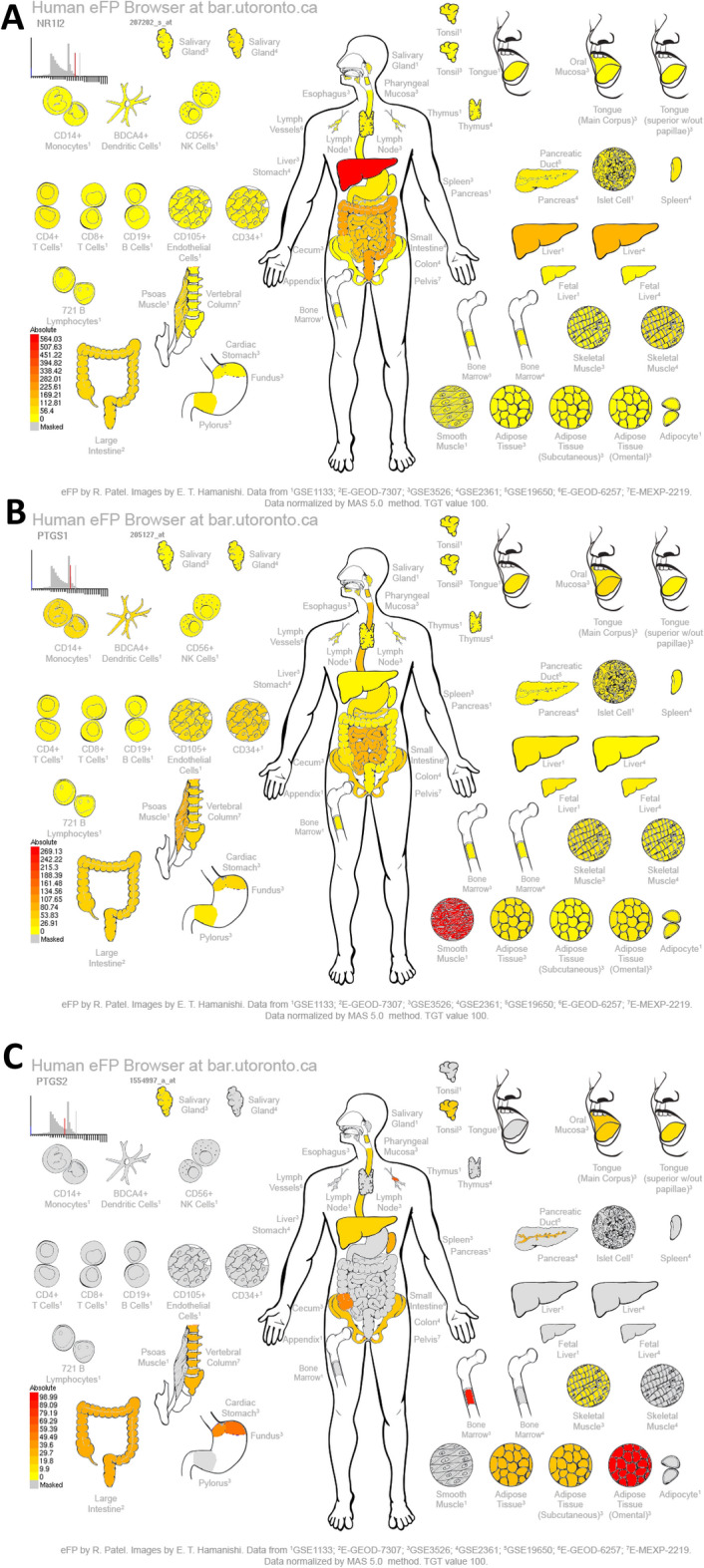

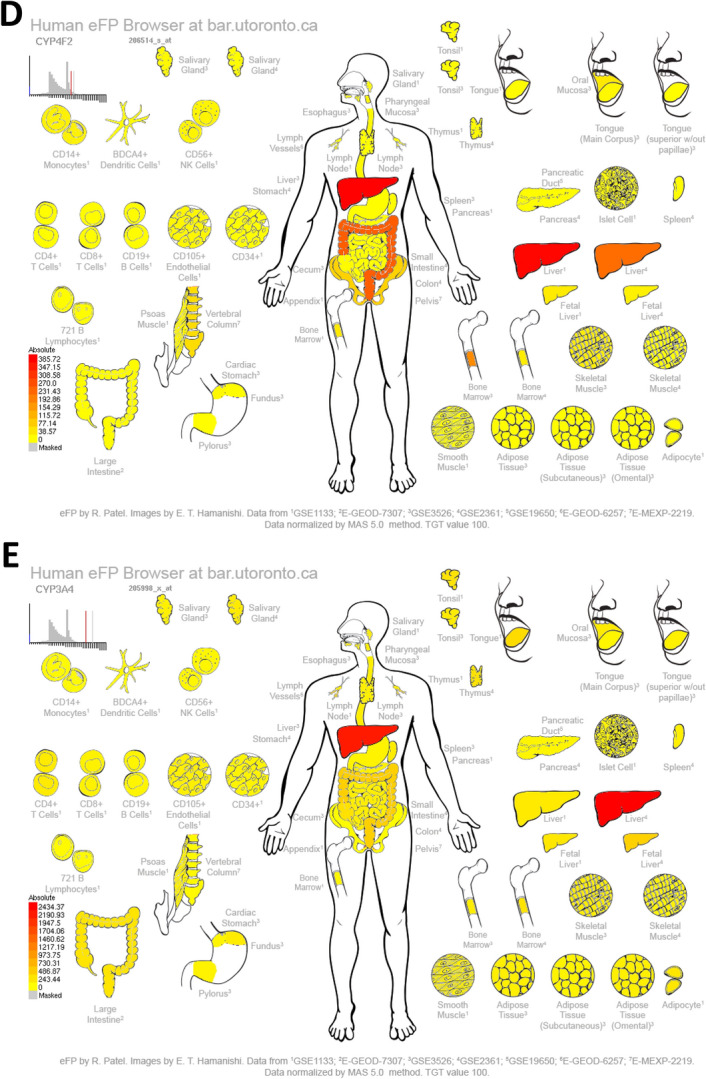
Analysis of (**A**): NR1I2, (**B**): PTGS1, (**C**): PTGS2, (**D**): CYP4F2, and (**E**): CYP3A4 expressions in the skeletal, immune, and digestive systems of a normal human.

The expressions of NR1I2, PTGS1, PTGS2, CYP4F2, and CYP3A4 were assessed to analyze the levels of core genes in the skeletal, immune, and digestive systems. The core gene expressions were different throughout the body. As shown in Fig. 13A–E, the expressions of NR1I2, PTGS1, CYP4F2, and CYP3A4 was ubiquitous in the skeletal, immune, and digestive systems of normal individuals. The expression of NR1I2 was the highest in the liver, while that of PTGS1 was the highest in smooth muscles. CYP4F2 and CYP3A4 were mainly expressed in the liver, mandible, and hindbrain. PTGS2 showed the highest expression in the omental adipose tissues of the skeletal, immune, and digestive systems, mainly in the human bone marrow, lymph node, cardiac stomach, and cecum.

### Molecular docking between the active ingredient of *Morchella esculenta*, EOYA, and its main anti-inflammatory targets

**Figure 14:**
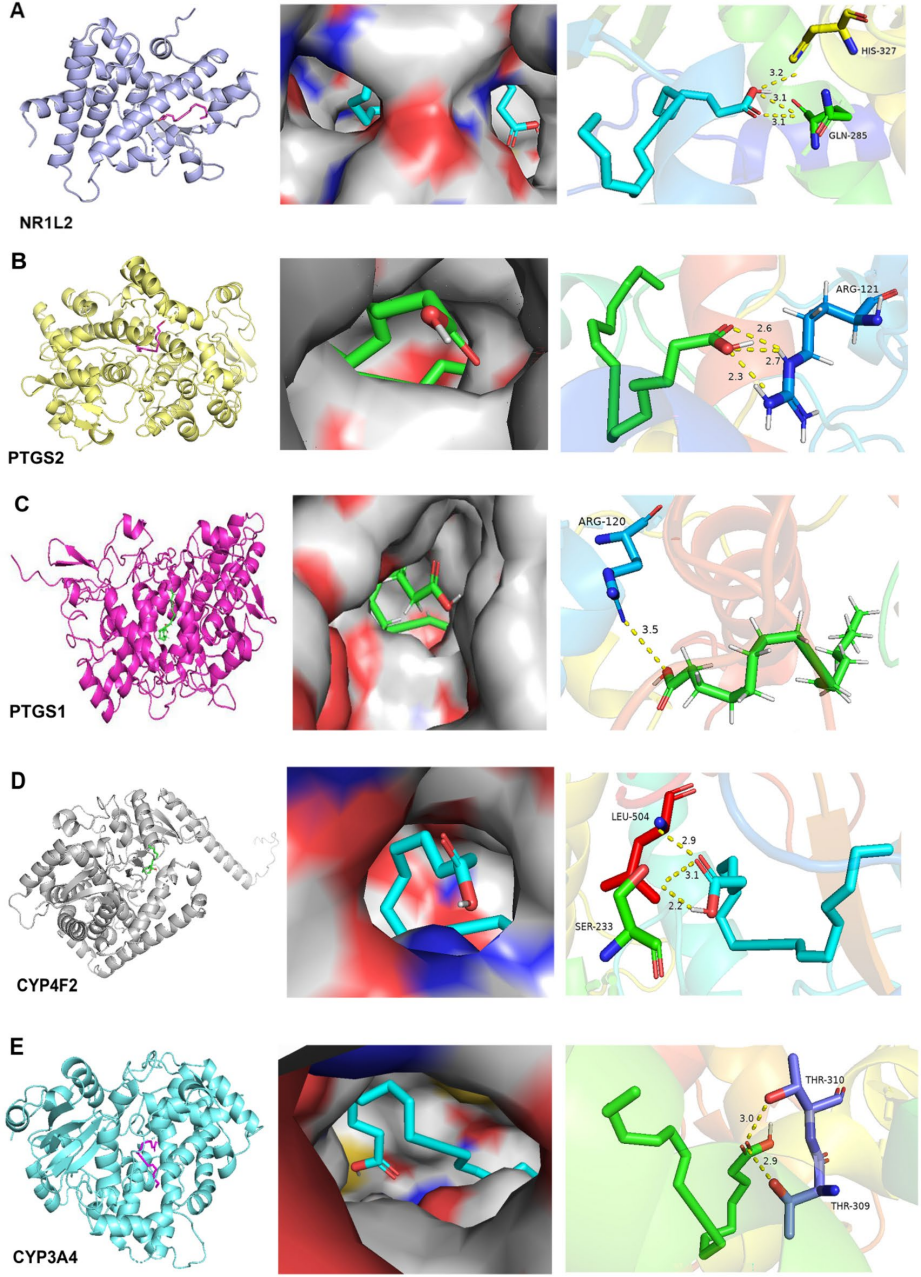
Molecular docking results for key anti-inflammatory targets of EOYA, the active ingredient of *Morchella esculenta* (**A**): NR1I2; (**B**): PTGS2; (**C**): PTGS1; (**D**): CYP4F2; (**E**): CYP3A4).

**Figure 15:**
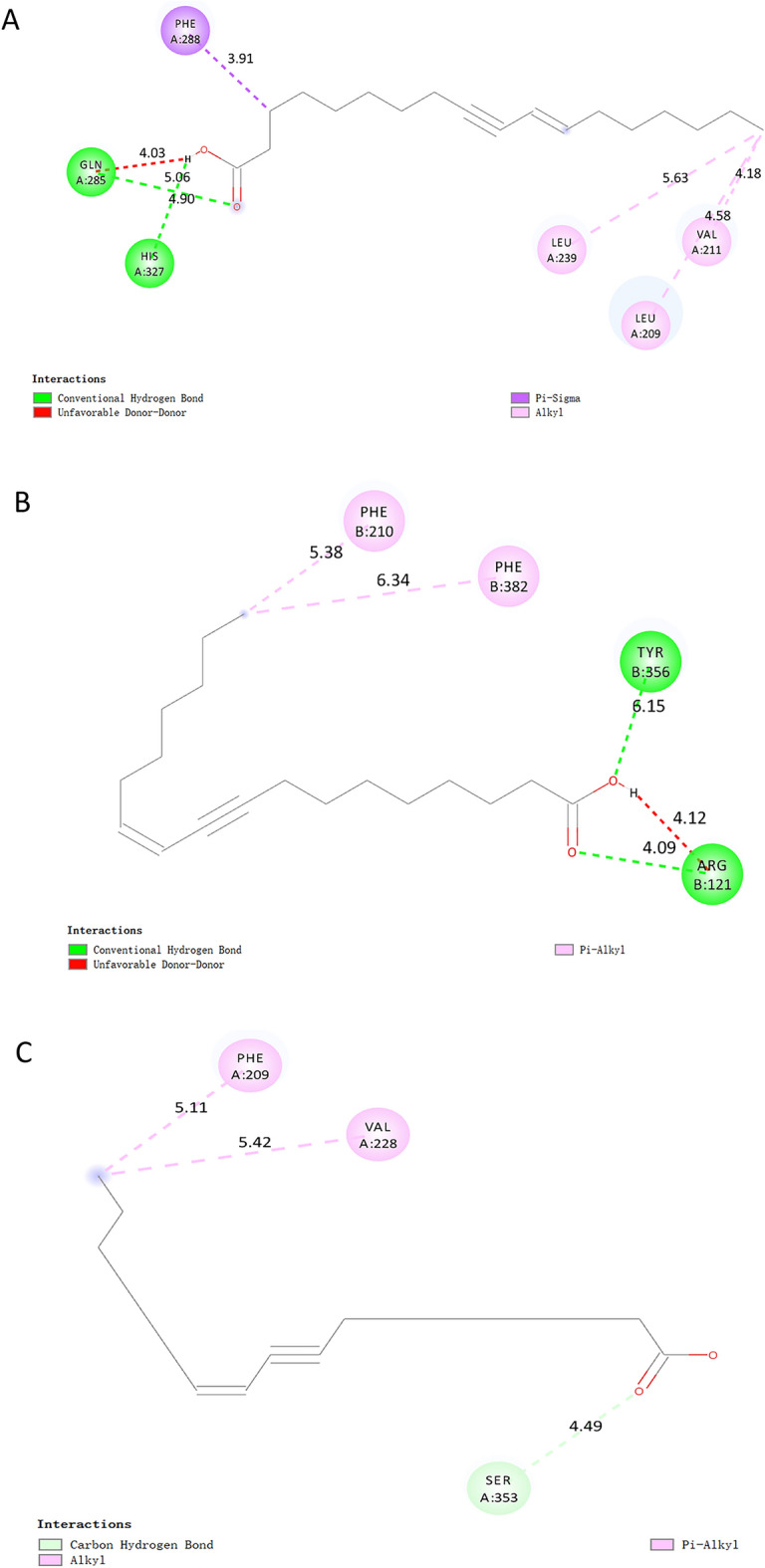

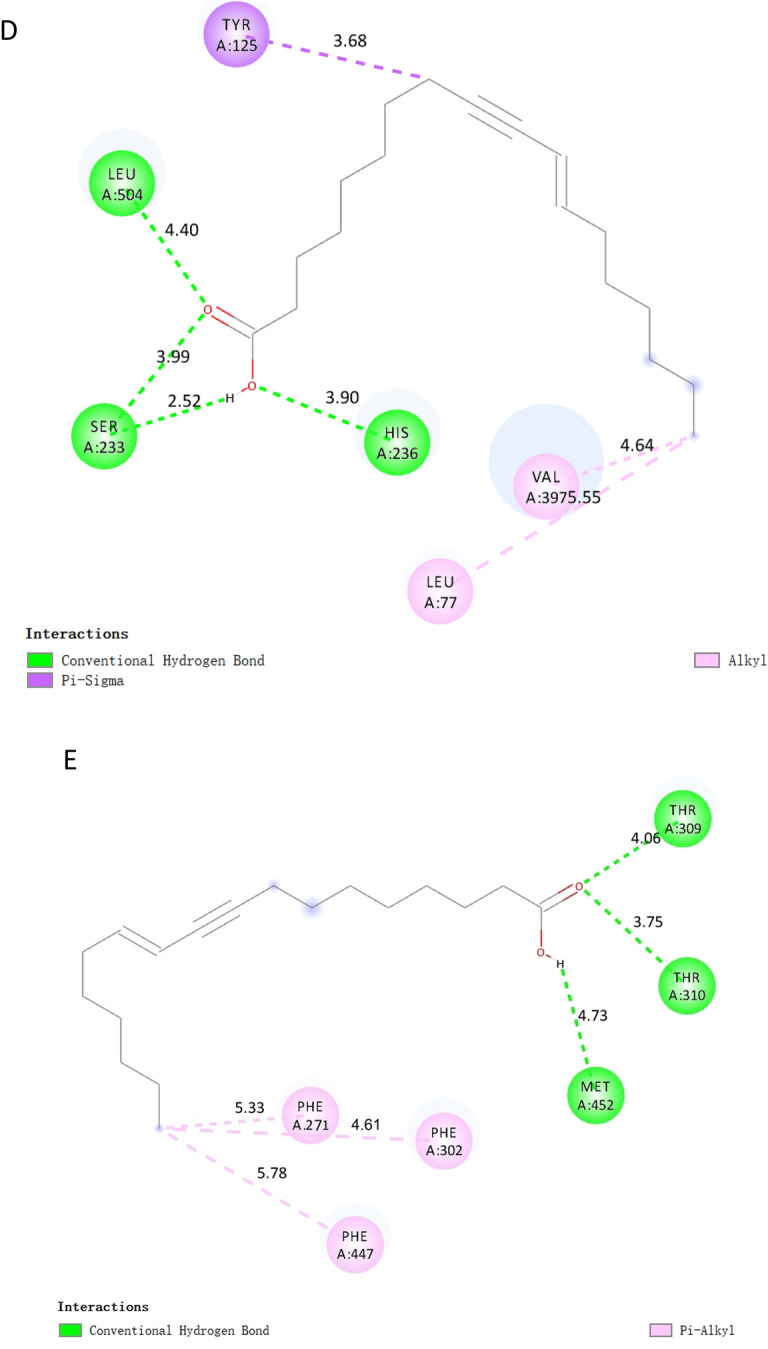
2D Structure of molecular docking for anti-inflammatory key targets of EOYA, the active ingredient of *Morchella esculenta* (**A**): NR1I2; (**B**): PTGS2; (**C**): PTGS1; (**D**): CYP4F2; (**E**): CYP3A4).

As shown in Table 2, the affinity between the nine target proteins and EOYA was less than − 5 kcal/mol, among which NR1I2, PTGS2, PTGS1, CYP3A4, and CYP4F2 (Figs. 14, 15) showed the highest affinity with − 7.0, − 7.0, − 6.8, − 6.8, 6.7 kcal/mol, respectively. These targets may be the key anti-inflammation targets of the active ingredient of *Morchella esculenta*, EOYA.

**Table 2:** Molecular docking parameters for anti-inflammatory key targets of EOYA, the active ingredient of *Morchella esculenta.*

Target	PDB ID	Coordinate	Binding energy (kcal/mol)	EOYA docking amino acid residues
NR1I2	1m13	X = 14.129; Y = 73.622; Z = 1.620	− 7.0	LEU206 LEU209 LYS210 VAL211 PRO227 ILE236 LEU239 LEU240 MET243 GLN285 PHE288 TRP299 CYS301 TYR306 MET323 HIS327
PTGS2	5f1a	X = 28.162; Y = 30.726; Z = 58.434	− 7.0	VAL117 ARG121 PHE206 THR207 PHE210 VAL229 VAL345 TYR349 VAL350 LEU353 SER354 TYR356 PHE382 TYR386 PHE519 MET523 VAL524 GLY527 ALA528 PHE530 SER531 LEU532 GLY534 LEU535
PTGS1	6Y3C	X = − 44.039; Y = 58.118; Z = 8.457	− 6.8	VAL116 ARG120 PHE205 PHE209 GLY227 VAL228 VAL344 TYR348 VAL349 LEU352 SER353 TYR355 LEU359 ASN375 ILE377 PHE381 TYR385 TRP387 PHE518 MET522 ILE523 GLY526 ALA527 PHE529 SER530 LEU531 GLY533 LEU534
CYP4F2	AF_AFP78329F1	X = 3.494; Y = − 1.562; Z = − 6.331	− 6.8	HIS63 GLN64 GLY65 MET66 VAL67 ASN68 LEU77 PHE88 VAL90 SER100 PHE124 TYR125 LEU128 HIS236 HIS237 VAL397 ILE398 SER399 LEU421 SER423
CYP3A4	4d6z	X = 13.853; Y = 34.772; Z = − 9.141	− 6.7	PHE137 ILE184 THR187 SER188 PHE271 PHE302 ALA305 GLY306 THR309 THR310 VAL313 LEU364 ILE369 ALA370 PRO434 PHE435 CYS442 GLY444 PHE447 ALA448 MET452
TLR4	2z65	X = 45.564; Y = 21.218; Z = 36.792	− 6.5	ILE32 VAL48 ILE52 LEU54 LEU61 ILE80 PHE119 PHE121 ILE124 TYR131 CYS133 PHE151 ILE153
PTPN11	4rdd	X = 0.586; Y = 5.909; Z = − 1.798	− 6.4	PRO312 PHE314 PRO323 LYS324 LYS325 SER326 TYR327 ASP473 ILE474 ASP477 ILE478 GLU481 LYS482 ASP487 ILE488 ASP489 LYS492 THR493
PLA2G4A	1jcy	X = 141.116; Y = 75.149; Z = 144.299;	− 6.3	MET1148 MET1342 PHE1343 ALA1344 ASP1345 TRP1346 MET1356 ALA1357 LYS1358 PRO1559 LEU1560 ARG1563 PRO1564 TRP1596 ASN1600 LEU1602
MAPK1	3i60	X = 10.445; Y = − 3.944; Z = 45.050	− 6.1	ILE29 GLU31 GLY32 ALA33 TYR34 GLY35 VAL37 ALA50 LYS52 ILE54 GLN103 ASP104 LEU105 MET106 GLU107 THR108 ASP109 SER151 LEU154 CYS164 ASP165

### MD simulation

**Figure 16:**
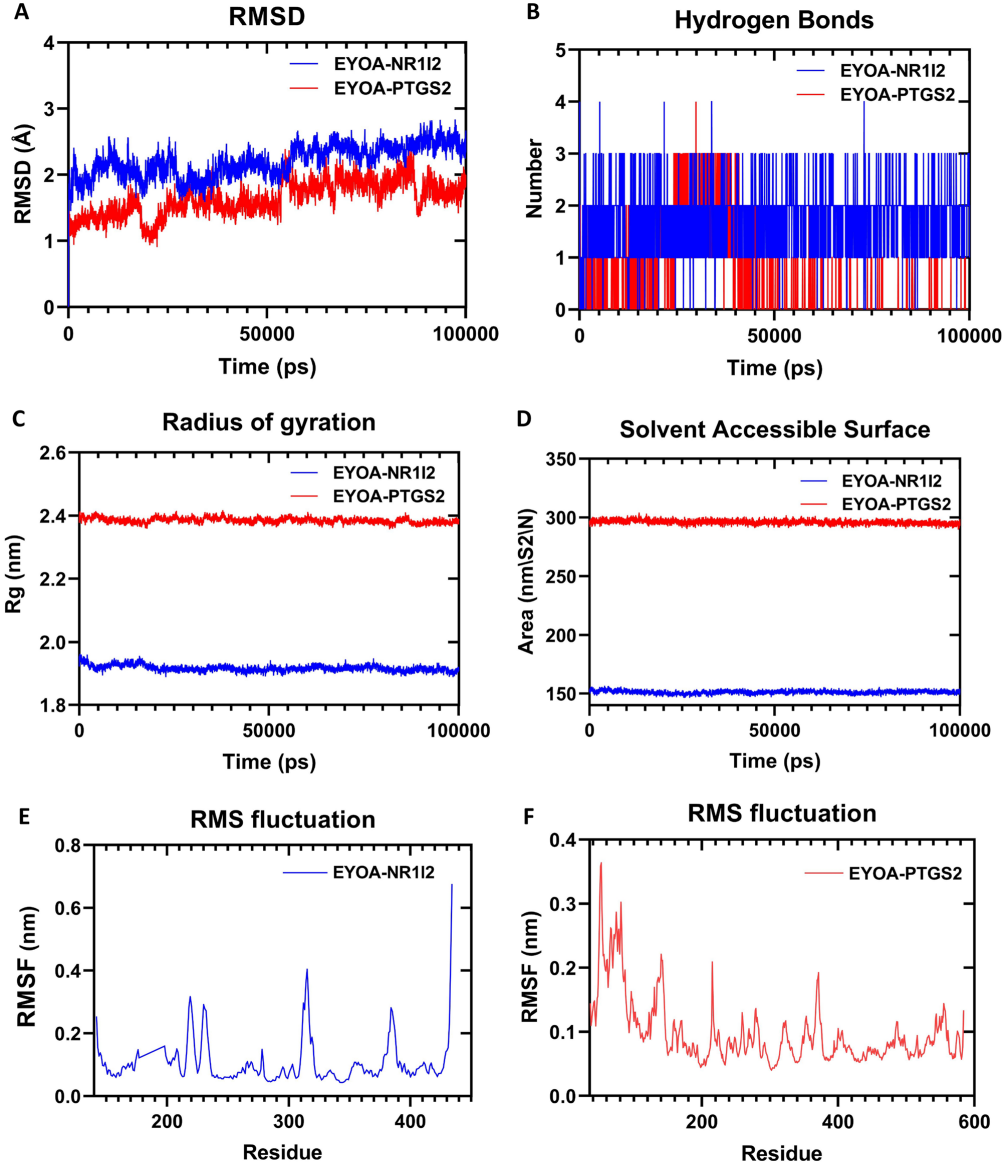
MD simulation results for key anti-inflammatory targets of EOYA, the active ingredient of *Morchella esculenta* (**A**): RMSD, (**B**): Number of hydrogen bonds, (**C**): Radius of gyration, (**D**): Solvent Accessible Surface, (**E**): The RMS fluctuation of EOYA-NR1I2, (**F**): The RMS fluctuation of EOYA-PTGS2). In all systerm, the color indicates-NR1l2 protein (blue), PTGS2 protein (red).

The top two best categorized docked complexes (EYOA-NR1I2 and EYOA-PTGS2) were additionally considered for molecular dynamic simulations to validate the network pharmacology and molecular docking results (Fig. 16). RMSD analysis of the protein give insights into its structural conformation during the simulations, providing an indication of the stability of the protein and whether the simulation has equilibrated. The results showed RMSD was less than 2.5 Å for two complexes until the end of the simulations, indicating that the complexes were overall stable, and it demonstrated that ETOA forms a stable complex with NR1I2 and PTGS2.RMSF was calculated to survey the fluctuation of every amino acid of the ligand–protein complex.In an RMSF plot, the peak indicates which region of the protein fluctuates most during the simulation, while lower RMSF values represent smaller conformational change.The RMSF values of the amino acid residues in loop regions were found to highly fluctuate in EYOA-NR1I2 model (142–434). The high value of RMSF fluctuation denotes strong variation of protein structure over all MD simulation. This is mainly due to strand breaks of amino acid from this portion of the protein. The EYOA-PTGS2 occurred some minor conformational changes that reflect the stability of the complex. For a more detailed characterization of hydrogen bond (H-bond) between protein and ligand atoms throughout MD trajectory, We counted the H-bonds for all systems during the course of 100 ns of simulation time. The result show that in the process of dynamics simulation, the hydrogen bonds of the complex always existed, and the number of hydrogen bonds were greater than or equal to 1 in EYOA-NR1I2 and EYOA-PTGS2 complex. These results indicate that small molecules form stable complex with proteins. Furthermore, no significant change in SASA for two proteins was observed, indicating that no significant part of the proteins was exposed to water, and the structure remained compact throughout the simulation time.We found that SASA analysis revealed a steady decline in SASA from 0 to 100 ns in EYOA-PTGS2 complex, indicating favourable binding and progressive protein tightening, and made the complex structure stable gradualy throughout the simulation.SASA also presents small decline from 0 to 20 ns upon ligand binding in EYOA-NR1I2, It might be that small molecules, after constantlycolliding with the active site in the protein pocket, found the rightconformation so as to reach the balance. The final results showed that EYOA did indeed bind well NR1I2 to PTGS2, and the complex has high stability.

## Discussion

*Morchella esculenta* is a fungus mushroom used both as medicine and food. It has the functions of invigorating the yang, resolving phlegm, tonifying the kidney, regulating the qi, invigorating the brain, pleen, and stomach, and aiding digestion. The role of *Morchella esculenta* in the arachidonic acid metabolic pathway has been studied. For instance, a specific LOX activity was determined from the crude extract of *Morchella esculenta*, and the affinity of LOX activity to the substrate, arachidonic acid, was strong (83%)^27^. The extracellular vesicles of *Morchella esculenta* contain some specific lipids, miRNAs, and proteins, which can inhibit LPS-induced inflammation by attenuating the production of ROS and reducing the phosphorylation levels in the p38 MAPK signaling pathway^28^. The active extract of *Morchella esculenta* can inhibit the expression of LPS-induced COX, and significantly inhibit the activity of NF-κB, indicating its preventive effect on inflammation^29^. To further analyze the anti-inflammatory mechanisms of action of *Morchella esculenta*, we then applied a network pharmacological method. However, the previous TCM network pharmacological research predicted drug targets through some databases only. This may be not fully consistent with the actual target of *Morchella esculenta*. To overcome these shortcomings, we use the 6 differents inflammation GEO database to provided more compelling evidence. Our results showed that we obtained one compounds EYOA derived from *Morchella esculenta* though the NPASS database, we searched multiple compound target databases and disease target databases and identified 43 anti-inflammatory targets of EOYA. According to the network pharmacology analysis, EOYA mainly played an anti-inflammatory role through arachidonic acid metabolism, vascular endothelial growth factor signaling pathway, and sheath phosphorus signal transduction pathway.

**Figure 17:**
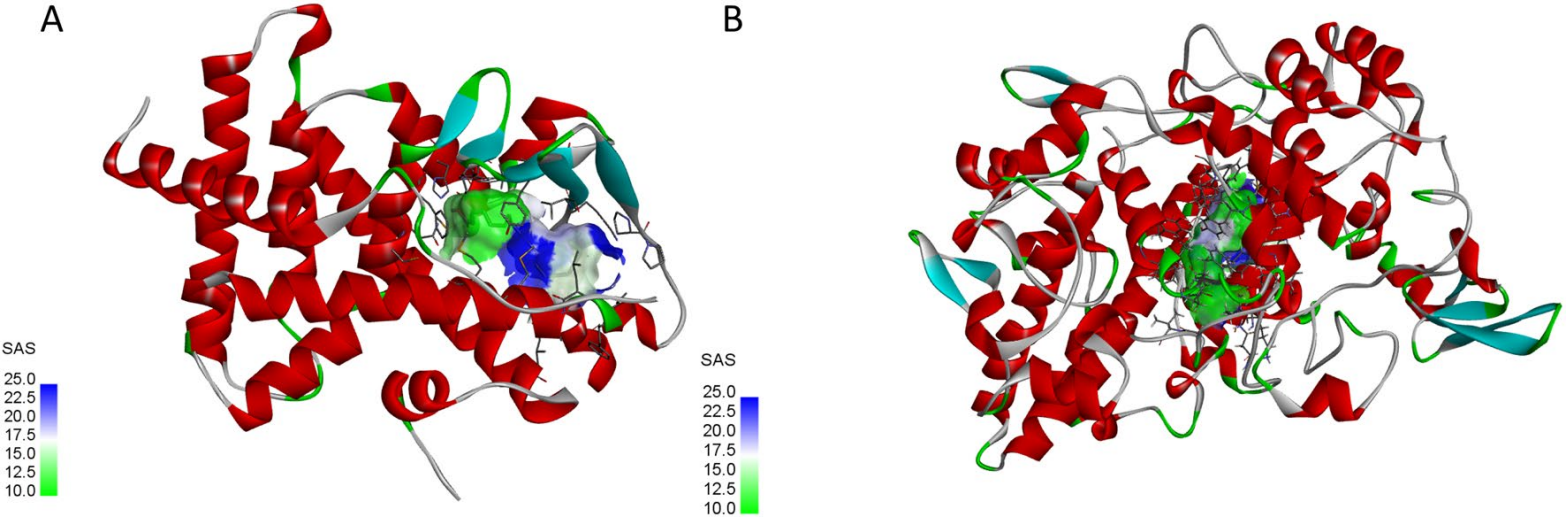
Active sites of docking between active components (EOYA) of *Morchella esculenta* and anti-inflammatory core targets. (**A**): EOYA-NR1I2; (**B**): EOYA-PTGS2).

9 potential targets were chosen as core targets based on the ‘Degree’, ‘Betweenness’ and ‘Closeness’ values in the PPI network, NR1I2, PTGS1, PTGS2, CYP4F2, CYP3A4, TLR4, MAPK1, PLA2G4A, and PTPN11. we expect to screen protein targets related to inflammation using 6 GEO databases related to inflammatory, Clinically relevant gene association networks obtained from these inflammation database. we obtained 5 tagerts significant differences with inflammation, including as NR1I2, PTGS1, PTGS2, CYP4F2 and CYP3A4. By using Autodock software EYOA is docked with 5 protein targets. The results show that all 5 proteins can bind well to EYOA. and the best characterized of these are NR1I2 and PTGS2. EYOA can form the strong interactions with the NR1I2 active site HIS 327 and GLN285, the PTGS2 active site ARG121. The studies of molecular dynamics simulations were carried out to understand more deeply the modes of interaction of the selected EYOA with the target proteins.The simulation result showed EYOA can form strong hydrogen bond interactions with the NR1I2 active site HIS 327 and GLN285, the PTGS2 active site ARG121 and TRY356, which was the main interaction force of EYOA bound with the active site of NR1I2 and PTGS2. The RMSD, RMSF, RG, hydrogen bond, and SASA define the complexes and show that they are more stable (Fig. 17). The analysis results of GEO dataset validating inflammatory core targets show that different types of inflammation have different significantly different targets, which may be related to the location of inflammation. But it also shows the application potential of EYOA in different inflammation.

Chronic inflammation is caused by various stimuli from natural immune cells through different pathogen-related modes. Its reaction process is continuous and complex, and these are mainly transmitted through the NF-κB signaling pathway^30^. The continuously increasing NF-κB activity can accelerate the aging of certain immune cells, leading to an increase in the levels of related inflammatory factors^31^. Simultaneously, the increase in the levels of reactive oxygen species (ROS) from mitochondria and cytoplasm and defective antioxidant protection result in continually altered redox metabolism^32^, and this redox imbalance changes the signaling response to growth, nutrition factors, cytokines, and chemokines, eventually leading to cell aging and chronic inflammation^33^. To date, hormone drugs are commonly used to treat inflammation. Dexamethasone is a glucocorticoid used to treat inflammatory diseases; it has been used for more than 50 years but its various side effects have been confirmed, including hypertension, adrenal insufficiency, and hyperglycemia^34^. Therefore, it is necessary to identify drugs with significant anti-inflammatory effects and fewer side effects. For centuries, traditional Chinese medicine has been used to treat various diseases^35^.

At present, the metabolites of fungi have become the main source of anti-inflammatory lead compounds because of their novel structure, low toxicity and significant inhibitory effect^36^. Many new compounds have been obtained from plant endophytic fungi, which can inhibit the excessive production of endotoxin-induced NO and pro-inflammatory cytokines IL-6, tumor necrosis factor-α and monocyte chemoattractant protein-1 at both gene and protein levels^37^. The compounds isolated from algae-producing fungi could inhibit the excessive production of NO and pro-inflammatory cytokines in lipopolysaccharide-treated RAW264.7 macrophages in a dose-dependent manner without any cytotoxicity^38^. New polyketones were isolated from marine fungus Eutyellahoraria. Nitric oxide was induced by lipopolysaccharide in RAW264.7 macrophages and showed strong anti-inflammatory activity. When the concentration was 50.0 μg/mL, the inhibition rate was 54.9%^39^. Edible and medicinal fungi are natural products with effective anti-inflammatory and antiviral properties. During the SARS-CoV-2 pandemic, people often ingested, reducing the impact of SARS-CoV-2^40^. *Agaricus blazei Murrill* has the properties of anti-allergy, anti-infection, asthma and anti-tumor. In mouse model, it also has anti-inflammatory effect in patients with inflammatory bowel disease^41^.

Inflammation, accompanied by increased vascular expansion permeability and leukocyte exudation, is an acute reaction involving and mediated by several chemical factors. The importance of arachidonic acid metabolism lies in the fact that it can be metabolized by three different enzymes, namely cyclooxygenases (COXs, also known as PGG/H synthetases), lipoxygenases (LOXs), and cytochrome P450 (CYP) enzymes (ω-hydroxylase and cyclooxygenase)^42^ (Fig. 16), which have attracted extensive attention in the inflammatory processes and diseases^43^ Prostaglandins, the products of arachidonic acid metabolism, are inflammatory mediators. Inflammation stimulates arachidonic acid metabolism and releases metabolites, such as prostaglandins and leukotrienes, causing fever, pain, and vasodilation in patients. In clinical practice, the most common manifestations of inflammatory reactions are redness, swelling, heat and pain, increased permeability, and leukocyte exudation.

PTGS1 is an essential enzyme responsible for prostaglandin synthesis^44^. PTGS1 can regulate peripheral vascular resistance, maintain renal blood flow, protect the gastric mucosa, and regulate platelet aggregation^45^. The expression of PTGS1 is related to several inflammatory pathways^46^. PTGS1 is distributed in microglia and plays a key role in regulating inflammation in the central nervous system^47^. Ursolic acid can treat autoimmune thyroiditis by inhibiting the mechanism and inflammatory pathways of the MALAT1/miR206/PTGS1 axis^48^. PTGS2 (or COX-2) is induced by hormones, growth factors, and inflammatory stimuli, and is considered an important source of prostaglandin formation in proliferative (such as cancer) and inflammatory diseases. CYP4F2 and CYP3A4 are enzymes of the cytochrome P450 family. The most prominent role of the CYP pathway is the metabolism of fat-soluble exogenous substances. The expression and activity of CYP are controlled by hormones, growth factors, and transcription factors^49^. The NR1I2-nuclear receptor subfamily, whose members are transcription factor coding proteins characterized by the presence of a DNA binding domain and a ligand binding domain. Its encoded protein is a transcriptional regulator of the cytochrome P450 gene CYP3A4.

**Figure 18:**
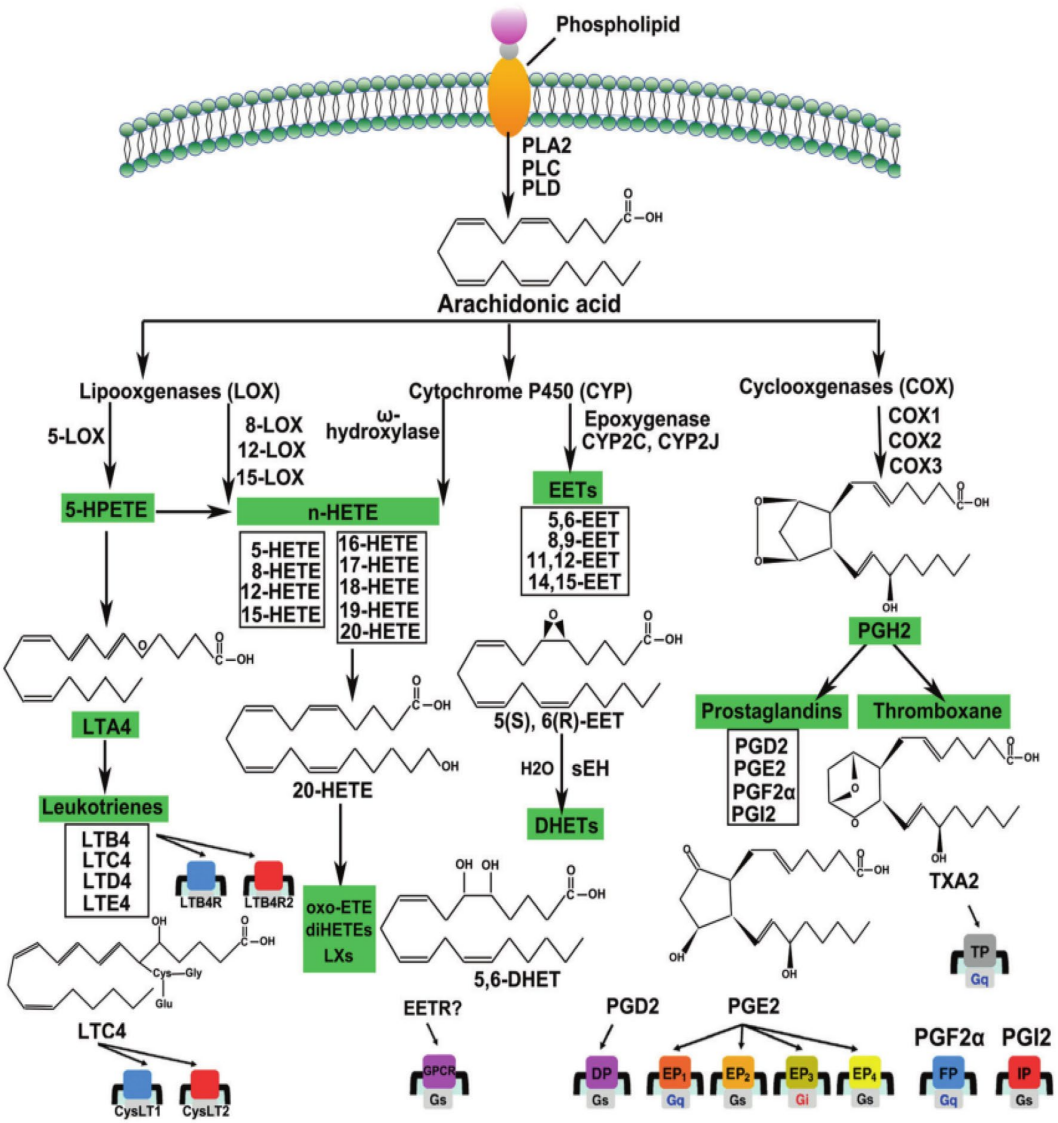
Three metabolic pathways involving arachidonic acid^42^.

The comparative and enrichment analyses of the effective components of *Morchella esculenta* showed that MAPK1, PTPN11, and TLR4 were involved in inflammation; PTGS2, PLA2G4A, CYP3A4, CYP4F2, PTGS1, and NR1I2 were involved in arachidonic acid-related pathways. CYP3A4, PTGS2, NR1I2, and PTGS1 were involved in the synthesis of prostaglandins. NR1I2, CYP3A4, and CYP4F2 participated in the cytochrome P450 pathway (Fig. 18). This showed that *Morchella esculenta* could participate in multiple metabolic pathways involving arachidonic acid, directly or indirectly regulate the activity of prostaglandin endoperoxide synthetase, and inhibit the release of prostaglandins, thereby reducing inflammation.

## Conclusion

In summary, *Morchella esculenta* has a multi-target pharmacological effect in the treatment of inflammation through the arachidonic acid metabolism pathway, which is expected to shed new insights into the treatment of inflammation. Herein, the network pharmacological strategy was used to study the multi-pathway and multi-target mechanisms of the active ingredient of *Morchella esculenta*, EOYA, in the prevention and treatment of inflammation. *Morchella esculenta* may be effective for the treatment of inflammation and provide a new direction for addressing the current shortcomings in treating inflammation.

## Data Availability

The data used to support the findings of this study are available from the corresponding authors upon reasonable request.

## Abbreviations

CYP3A4: Cytochrome p450 family 3 subfamily a polypeptide 4
CYP4F2: Phylloquinone omega-hydroxylase CYP4F2
EOYA: (E)-Octadec-11-En-9-Ynoic Acid
NR1I2: Nuclear receptor subfamily 1 group I member 2
PLA2G4A: Cytosolic phospholipase A2
PTGS1: Prostaglandin G/H synthase 1
PTGS2: Prostaglandin G/H synthase 2
PTPN11: Tyrosine-protein phosphatase non-receptor type 11
TLR4: Toll-like receptor 4
